# Prospective study of antidepressant treatment of psychiatric patients with depressive disorders: treatment adequacy and outcomes

**DOI:** 10.1186/s12888-023-05390-8

**Published:** 2023-11-28

**Authors:** Johanna von Knorring, Ilya Baryshnikov, Pekka Jylhä, Tiina Talaslahti, Martti Heikkinen, Erkki Isometsä

**Affiliations:** Department of Psychiatry, University of Helsinki and Helsinki University Hospital, P.O. Box 22, Helsinki, FI-00014 Finland

**Keywords:** Depression, Psychiatric care, Antidepressant, Treatment adequacy, Treatment outcome

## Abstract

**Background:**

Despite numerous national depression care guidelines (DCGs), suboptimal antidepressant treatment may occur. We examined DCG concordance and depression treatment outcomes in psychiatric settings.

**Methods:**

We evaluated treatment received and outcomes of 128 psychiatric out- and inpatients participating in the PEGAD (Pharmacoepidemiology and Pharmacogenetics of Antidepressant Treatment for Depressive Disorders) study at baseline, two weeks, and eight weeks using interviews and questionnaires. Inclusion criteria were ICD-10 diagnosis of a depressive disorder, a Patient Health Questionnaire-9 symptom (PHQ-9) score ≥ 10, and a new antidepressant prescribed. The primary outcome of the study was within-individual change in PHQ-9 scores.

**Results:**

At baseline, patients had predominately recurrent (83%) and in 19% treatment-resistant depression (TRD). The median preceding duration of the current episode was 6.5 months. At eight weeks, 85% of the patients (n = 107) used a DCG-concordant antidepressant dose. However, due to the scarcity of antidepressant combinations and augmentations, fewer TRD than non-TRD patients (25% vs. 84%, *p* < 0.005) received adequate antidepressant treatment. Additionally, one-third of the patients received inadequate follow-up. Overall, only 53% received treatment compatible with DCG recommendations for adequate pharmacotherapy and follow-up. The mean decline in PHQ-9 scores (-3.8 ± SD 5.7) was significant (*p* < 0.0005). Nearly 40% of the patients reached a subthreshold level of depression (PHQ-9 < 10), predicted by a lower baseline PHQ-9 score, recurrent depression, and female sex. However, 45% experienced no significant clinical improvement (PHQ-9 score reduction < 20%).

**Conclusions:**

Our findings suggest that inadequate treatment continues to occur in psychiatric care settings, particularly for TRD patients.

## Introduction

Depression treatment should strive for full remission. The essential treatment option of antidepressants (ADs) is endorsed by numerous national depression care guidelines (DCGs), e.g. [1–4], particularly in moderate to severe depression, as robust evidence indicates their effectiveness and safety [5]. AD monotherapy is an evidence-based treatment option for acute depression and relapse prevention, which, following Finnish DCG recommendations [6], is predominately carried out in primary care. Patients with psychotic or treatment-resistant depression (TRD, defined as depression not responding to two or more adequately conducted AD monotherapy trials; [7]) and significant psychiatric comorbidity, functional disability, or suicidality should be referred to psychiatric care as they often require more complex pharmacotherapy, including AD combinations and augmentation with lithium or an antipsychotic [8–14]. To facilitate therapeutic decisions based on outcome predictors and observed treatment responses [15–17], DCGs endorse measurement-based care (MBC), i.e., routine monitoring of clinician- or patient-rated depression measures [18–20]. Thus, MBC provides clinicians with evidence-based tools for individualized depression treatment, additionally associated with better outcomes [19–21].

However, DCG-discordant or individually sub-optimized depression treatment is still an acknowledged problem in primary and psychiatric care settings [22–28]. Vigo et al. [29] concluded that only 10% of patients with major depressive disorder (MDD) received an adequate combination and implementation of pharmacotherapy and psychotherapy. Specifically, the main shortcomings of pharmacotherapy were underutilization and inadequate clinical monitoring of responses and side effects. A nationally representative survey study from the USA [30] found that less than one-third of non-remitted, AD-treated patients received augmentation treatment. One naturalistic European study [31] evaluating DCG adherence in outpatient care noted a scarcity of AD dose and medication changes, regardless of treatment outcome. Moreover, primary and specialized psychiatric care patients have been described as unexpectedly similar in depressive symptoms and depression severity [27, 32, 33], indicating possible clinical practice conflicting with DCG recommendations concerning referral to specialized care. Previously reported rates of depression treatment adequacy in psychiatric care have shown a wide variation between 31.0% and 94.4% [23, 24, 34–36]. However, there are very few studies investigating the concordance of treatments provided in psychiatric settings with DCG recommendations during the current era of guidelines and widespread AD use.

Earlier Finnish studies have also shown quality-of-care problems in depression treatment, e.g., treatment initiation delays, suboptimal treatment intensity, continuity challenges, and an indistinct division of labour between primary and psychiatric care [37–40]. Lähteenvuo et al. [41] recently reported on a nationwide register-based cohort that AD monotherapy was the most frequently initiated treatment, even among those TRD patients progressing to a fifth treatment trial after four previous monotherapies. This finding indicates likely non-adherence to DCG recommendations, as pharmacological combination or augmentation strategies are recommended after two failed monotherapy trials. Therefore, a more detailed study in a smaller sample is vital to broadening the understanding of current depression treatment practices and outcomes in psychiatric care settings. The Finnish DCG, first published in 2004, was recently updated in 2020. However, most studies published on the clinical practice of depression treatment in the Finnish public health care system date back more than a decade. Little is known about how treatment practices may have changed over the years. Additionally, when considering factors impacting depression care practice, we cannot ignore the 40% reduction in Finnish psychiatric hospital beds in recent years [42] and the increasing shortage of psychiatrists and other healthcare professionals in the public sector.

In this observational eight-week follow-up study, we aimed to examine Finnish DCG adherence in the psychiatric care settings of Helsinki University Hospital, Finland’s largest hospital district. Specifically, we aimed to describe (1) the clinical characteristics, prior course of illness, and treatment history of patients currently referred to psychiatric care, (2) the treatment received, focusing on AD use, and (3) the short-term treatment outcome. We expected treatment practice to align with the current recommendations, including appropriate AD dosage, treatment duration, and follow-up.

## Methods

### Study design and setting

This eight-week observational prospective cohort study is part of the PEGAD (Pharmacoepidemiology and pharmacogenetics of antidepressant treatment for depressive disorders) project, conducted within Helsinki University Hospital´s divisions of Acute Psychiatry, Mood Disorders, and Geropsychiatry. The Helsinki University Hospital catchment area provides adult psychiatric out- and inpatient services to Espoo, Kauniainen, Kirkkonummi, Vantaa, and Kerava, and geropsychiatric services to Helsinki and neighbouring cities, facilities from which clinicians recruited study patients between August 2018 and November 2019. The Ethics Committee of Helsinki University Hospital and the Finnish Medicines Agency (FIMEA) approved the study protocol. All recruited patients gave written informed consent. The study was based on clinical diagnoses by attending psychiatrists responsible for providing patients’ usual care. During follow-up patients received treatment as usual for their depressive disorder.

### Inclusion and exclusion criteria

The inclusion criteria were as follows: (1) an ICD-10 (International Statistical Classification of Diseases and Related Health Problems 10th Revision) [43] diagnosis of Depressive episode (F32) or Recurrent depressive disorder (F33), (2) a Patient Health Questionnaire-9 (PHQ-9) [44] score ≥ 10, (3) a new AD prescribed, and (4) age ≥ 18 years. The exclusion criteria were (1) a principal clinical diagnosis other than depression, (2) current psychotic symptoms, (3) immediate suicide risk, and (4) involuntary hospitalization.

### Evaluation and scales

We evaluated patients at three time points: baseline, two weeks, and eight weeks.

*At baseline*, research nurses collected sociodemographic and clinical data using interviews and instructed patients to answer the self-report questionnaires. Symptom and treatment history of current major depressive episode (MDE) was based on patients’ recollection. Patients were asked structured questions to estimate the time points for initial signs and symptoms reaching the level of clinical depression. For each AD reportedly used before study participation, we enquired when the patients had used it, the duration of its use, its highest dosage, and the reason for its discontinuation.

The Columbia–Suicide Severity Rating Scale (C-SSRS) [45] was used to identify and rate suicidal ideation and behaviour occurring after the initial screening and admittance to the study.

The self-report scales included the PHQ-9, the Overall Anxiety and Impairment Scale (OASIS) [46], the Mood Disorder Scale (MDQ) [47], the Snaith-Hamilton Pleasure scale (SHAPS) [48], the Sheehan Disability Scale (SDS) [49], the Alcohol Use Disorders Identification Test (AUDIT) [50], questions on illegal drug use, the Fagerström Test for Nicotine Dependence (FTND) [51], and the McLean Screening Instrument for Borderline Personality Disorder (MSI-BPD) [52].

*At two weeks*, patients received an email inquiry asking if they still used the AD described at baseline. Additionally, patients were asked to fill in the PHQ-9, OASIS, and MDQ.

A*t eight weeks*, research nurses conducted interviews concerning treatment received and patient adherence during follow-up. Drug use was specified by asking about all current psychiatric and somatic medication (dosage and time of last use) and psychiatric medication used during the preceding week. The patients also filled in the PHQ-9, OASIS, SHAPS, SDS, and MDQ.

### Primary outcome

The *primary outcome* of depression treatment was the within-individual change in PHQ-9 symptom scores between baseline and the eight-week time point. We also divided patients into groups according to baseline depression severity, examining the change in PHQ-9 symptom scores between baseline and the eight-week time point specifically in each group.

### Minimally adequate treatment

The overall treatment received during the study was classified as “minimally adequate” for non-TRD patients when the following criteria were met: (1) receiving AD for two months and having an adequate treatment dose (defined by the Finnish DCG) at the eight-week time point and (2) including at least two follow-up visits at the treating facility. “Minimally adequate” treatment for TRD patients had the additional criterion of receiving AD combination or pharmacological augmentation treatment.

### Statistical analysis

As the amount of missing symptom scale data was small (data available on request), we used the mean substitution method to address missing data. We used the Chi-square and Fisher Exact tests to examine associations between categorical variables and the Mann-Whitney test to compare continuous variables between the ICD-10 specific diagnosis groups. The effects of independent baseline variables on the likelihood of reaching a PHQ-9 value < 10 at the eight-week time point were examined using a multivariate logistic regression model. We performed all analyses using the SPSS program [53].

## Results

### Patient sampling

We excluded three of the 131 patients recruited for technical reasons, resulting in a baseline patient number of 128. Ninety-one patients (71.1%) provided data at two weeks and 107 (83.6%) at eight weeks. Most (n = 113; 88.3%) were outpatients at the time of recruitment, and the rest (n = 15; 11.7%) were inpatients. All follow-ups were conducted in outpatient care. A minority of participants (n = 16; 12.5%) were psychogeriatric patients (age > 65 years).

### Patient characteristics

The majority (n = 85; 66.4%) of the 128 patients were women, predominantly (n = 54; 63.5%) unmarried, separated, or widowed. Women had a higher educational level (p = 0.047) and were more likely to be a part of the active workforce (*p* < 0.001) than men (Table 1). Patients who dropped out after the baseline evaluation (n = 21) did not differ in their baseline clinical characteristics from those remaining in follow-up (data available on request).

**Table 1:** Clinicodemographic characteristics of patients with depression referred to psychiatric care (n = 128)

	Women n = 85 (%)	Men n = 43 (%)	Total, n = 128 (%)	p-value*
**Age** (mean)	38	39	38	
**Marital status**				0.130^d^
Married, in a registered partnership, cohabiting	31 (36.5)	10 (23.3)	41 (32)	
Unmarried, separated, widowed	54 (63.5)	33 (76.7)	87 (68)	
**Guardian of minors** ^**a**^	21 (24.7)	6 (14)	27 (21.1)	0.150^d^
**Housing type**				
Living alone (tested for living alone vs. living with others)	31 (36.5)	22 (51.2)	53 (41.4)	0.111^d^
Living with immediate family	51 (60)	17 (39.5)	68 (53.1)	
Homeless	0	1 (2.3)	1 (0.8)	
Other	3 (3.5)	3 (7)	6 (4.7)	
**Education (highest completed degree)**				
Basic (no high school or vocational training)	23 (27.1)	20 (46.5)	43 (33.6)	
Intermediate (high school or vocational school)	26 (30.6)	13 (30.2)	39 (30.5)	
High (higher vocational school, polytechnic, or university)	36 (42.4)	10 (23.3)	46 (35.9)	0.047^d^
**Work status**				
Employed	20 (23.5)	10 (23.3)	30 (23.4)	
Student	17 (20)	4 (9.3)	21 (16.4)	
Unemployed	5 (5.9)	10 (23.3)	15 (11.7)	
Retired, disability pension for medical reasons	13 (10.2)	6 (14)	19 (14.8)	
Sick leave	25 (29.4)	11 (25.6)	36 (28.1)	
Disability pension for psychiatric reasons (disability > 12 months)	1 (1.2)	1 (2.3)	2 (1.6)	
Parental leave, military, or non-military service	4 (4.7)	1 (2.3)	5 (3.9)	
Part of active workforce (including sick leave ≤ 12 months)	70 (82.4)	35 (81.4)	105 (82)	< 0.001^d^
**Own perceived working ability** ^**b**^				0.281^d^
Good	10 (11.8)	9 (20.9)	19 (14.8)	
Impaired	37 (43.5)	14 (32.6)	51 (39.8)	
Unable to work	29 (34.1)	16 (37.2)	45 (35.2)	
**Own perceived financial status**				0.149^d^
Good, adequate	49 (57.6)	19 (44.2)	68 (53.1)	
Fair, poor	36 (42.4)	24 (55.8)	60 (46.9)	
**Chronic medical conditions**	36 (42.4)	25 (58.1)	61 (47.7)	0.091^d^
**AUDIT ≥ 8**	21 (24.7)	9 (20.9)	30 (23.4)	0.227^d^
**Regular smoking**	20 (23.5)	9 (20.9)	29 (22.7)	0.110^d^
**Illegal drug use within 12 months** ^**c**^	6 (7.1)	5 (11.6)	11 (8.6)	0.508^e^

AUDIT, Alcohol Use Disorders Identification Test (scores ≥ 8 indicating harmful alcohol consumption)

*Men vs. women

^a^Data missing for 1 woman (1.2%)

^b^Data missing for 9 women (10.6%) and 4 men (9.3%)

^c^Data missing for 4 women (4.7%) and 1 man (2.3%)

^d^Chi-square test

^e^Fisher exact test

### Prior course of Illness

Most (n = 106; 82.8%) of the 128 patients were diagnosed with recurrent depression with a median of three episodes (Table 2). The median age for all patients’ first MDE was 17 years (range 6 to 83 years, mean 24 years). Patients had suffered from their current MDE for a median of 6.5 months, but 19.5% (n = 25) had a chronic index episode of two years or longer. Patients with recurrent depression reported a shorter duration of symptoms (median 5.5 months vs. 16.0 months, p = 0.005) and had sought treatment faster (median five months vs. six months, p = 0.048) than first-episode patients. First-episode depression was also associated with a likelihood of a chronic index episode (p = 0.014) but not with a more severe level of depression at baseline. TRD patients did not differ from others regarding the baseline severity of depression, anxiety symptoms, or functional impairment, nor were they associated with chronic medical problems (data available on request).

**Table 2:** Preceding course of depression and clinical and treatment characteristics of psychiatric patients with depression (n = 128)

Current ICD-10 diagnosis of depression for n = 128	
- F32 (Depressive episode): n (%)	22 (17.2)
- F33 (Recurrent depressive disorder): n (%)	106 (82.8)
**Age at first depressive episode** (years): Median age for n = 128 (IQR)^a^	17 (17)
- F32: Median age (IQR)	21 (17)
- F33: Median age (IQR)	16 (18)
**Total number of depressive episodes for patients with recurrent depression** (n = 106)	
- Number of depressive episodes: Median (IQR)	3 (2)
- Reached full recovery after previous depressive episode^b^: Median (IQR)	85 (80.2)
**Current depressive episode** for n = 128	
- Psychiatric caretaking institution at recruitment	
Outpatient clinic: n (%)	113 (88.3)
Hospital ward: n (%)	15 (11.7)
Time from first symptom onset (months): Median (IQR)	14.5 (26.8)
- Time from onset of depressive episode (months): Median (IQR)	6.5 (9.8)
- Time from first consultation (months): Median (IQR)^c^	5 (10)
- Patients using ADs before referral to current treatment: n (%)	91 (71.1)
- Patients with previous AD trials (n = 91) for their current depression episode	
1 previous AD trial: n (%)	46 (50.5)
2 previous AD trials: n (%)	25 (27.5)
3–6 previous AD trials: n (%)	20 (22)
- Psychotherapy received before referral to current treatment: n = 128 (%)	27 (21.1)

IQR, interquartile range; AD, antidepressant

^a^Missing data for 2/128 patients (1.6%)

^b^Missing data for 1/106 patients with recurrent depression (0.9%)

^c^Missing data for 1/128 patients (0.8%)

### Treatment received for current depressive episode (index episode) before baseline

Ninety-one (71.1%) of the 128 patients had used ADs for their current episode before study participation, half of whom (n = 46; 50.5%) had only one AD, with no significant difference between recurrent and first-episode depression patient groups. Commonly used ADs were escitalopram (n = 43), bupropion (n = 23), mirtazapine (n = 21), and venlafaxine (n = 17). Among the 128 patients, 18.8% (n = 24) had not responded to at least two adequately conducted AD trials before entering the study and were identified as TRD patients.

Before baseline, 21.1% (n = 27) of the patients attended psychotherapy, and over half (n = 72; 56.3%) reported attending supportive discussions with a health care professional. However, 25% (n = 32) had no regular treatment contact for their current depression before referral to psychiatric care.

### Treatment received during the study

We present an overview of the treatment received during follow-up in Table 3. In accordance with the inclusion criteria, all 128 study patients were prescribed an AD at baseline. The most prescribed ADs were escitalopram (n = 22), bupropion and venlafaxine (n = 18 each), duloxetine (n = 17), and sertraline (n = 16). At eight weeks, 90.7% (n = 97) of the 107 patients remaining in the follow-up reported currently using ADs, 93.8% (n = 91) of whom had a dosage within the therapeutic range recommended by the Finnish DCG. Thirty (30.9%) of the 97 patients using ADs used an AD combination. Quetiapine was the only atypical antipsychotic used for AD augmentation, prescribed to nine patients (9.3%), one of whom also received augmentation with lamotrigine.

**Table 3:** Treatment of psychiatric patients with depressive disorders during an eight-week follow-up (n = 107)

	All patients n = 107 N (%)^a^	TRD n = 20^b^ N (%)	non-TRD n = 87 N (%)	p-value^*^
**Follow-up appointments**				
- Psychiatrist (A)	73 (68.2)	13 (65)	60 (69)	0.963^c^
- Other health care worker (B)	74 (69.2)	13 (65)	61 (70.1)	0.884^c^
- Any appointment overall (A, B, or A + B)	94 (87.9)	17 (85)	77 (88.5)	1^d^
- No follow-up appointments	12 (11.2)	2 (10)	10 (11.5)	
Psychotherapy and other treatment sessions				
- Psychotherapy	14 (13.1)	4 (20)	10 (11.5)	
- Supportive meetings at another service provider	64 (59.8)	14 (70)	50 (57.5)	
- Family meetings	1 (0.9)	0	1 (1.1)	
- Group counselling	5 (4.7)	0	5 (5.7)	
- Visits at occupational health care unit	2 (1.9)	1 (5)	1 (1.1)	
**Use of antidepressants (ADs) at eight weeks**				
Patients using ADs (n_tot_ for AD use):	97 (90.7)	18 (90)	79 (90.8)	1^d^
- using AD monotherapy only	67 (69.1)	14 (77.8)	53 (67.1)	
- using a combination of ADs	30 (30.9)	4 (22.2)	26 (32.9)	0.439^c^
- AD dosage compatible with Finnish DCG recommendations for a therapeutic dose	91 (93.8)	18 (100)	73 (92.4)	0.298^d^
Did the patient continue using the index AD prescribed at BL until eight weeks?				
- Yes, used index AD as only AD	50 (51.5)	10 (55.6)	40 (50.6)	
- Yes, used index AD in combination with other AD(s)	27 (25.2)	3 (16.7)	24 (30.4)	
- No, index AD switched to another AD, which was used alone or combined with other AD(s)	15 (15.5)	4 (22.2)	11 (13.2)	
- No, index AD terminated, patient continued using previously prescribed AD(s)	5 (4.7)	1 (5.6)	4 (5.1)	
- No, all ADs terminated	8 (7.5)	1 (5.6)	7 (8.9)	
- No, never started using the index AD and not using any other AD	1 (0.9)	0	1 (1.3)	
**Augmentation pharmacotherapy (combined with ADs)**				1^d^
- Atypical antipsychotic (AA) (quetiapine, minimum 50 mg/day)	9 (9.3)	1 (5.6)	8 (10.1)	1^d^
- Mood stabilizer (lamotrigine)	1 (1.0)	0	1 (1.3)	
**Monotherapy: using one AD or one AA only** (quetiapine ≥ 50 mg/day)	62 (58.0)			
**ECT during follow-up** (n = 107)	1 (0.9)	0	1 (1.5)	
**New hospitalization during follow-up** (n = 107)	2 (1.9)	0	2 (2.3)	

AD, antidepressant; index AD, antidepressant assigned to study patient at baseline; AA, atypical antipsychotic; ECT, electroconvulsive therapy; BL, baseline; TRD, patient classified with treatment-resistant depression at baseline; non-TRD, patient classified as not having treatment-resistant depression at baseline

^a^Missing data for 1/107 patients (0.9%)

^b^Missing data for 1/20 TRD patients (5%)

^c^Chi-square test

^d^Fisher exact test

*TRD vs. non-TRD

Of the 107 patients finishing the study, roughly 1/3 had no or only one follow-up visit, 1/3 had two visits, and 1/3 had more than two visits (median for all patients was 2 visits, range 0 to 24 visits). Psychiatrists met the study patients on average once during the follow-up, but 30.8% (n = 33) of patients did not meet their treating psychiatrist after the initial meeting. However, three patients had multiple follow-up visits (18, 20, and 24 visits), mainly with a psychiatric nurse, together constituting 17.2% of all follow-up visits among study patients. All three patients suffered recurrent depression, classified as severe for one, moderate for one, and mild for one. None of these three patients reported acute suicidality. The TRD and non-TRD patients did not differ in the frequency of follow-up visits.

Altogether 53.3% of all patients received overall treatment classified as “minimally adequate”, including adequate pharmacological treatment and follow-up. As the Finnish DCG recommendations differ for the TRD and non-TRD patients, we examined the adequacy of treatment received separately for these two patient groups.

### Treatment of non-TRD patients

Eighty-seven non-TRD patients (83.7%) finished the study, at which point most (n = 79; 90.8%) used ADs. Of the 79 non-TRD patients using ADs, 92.4% (n = 73) had a DCG-concordant treatment dosage, 32.9% (n = 26) used an AD combination, and 10.1% (n = 8) received AD augmentation with quetiapine, one additionally with lamotrigine. Furthermore, one non-TRD patient received electroconvulsive therapy (ECT) during follow-up.

Accounting for suboptimal follow-up, while 83.9% (n = 73) of all non-TRD patients completing the study received DCG-recommended AD treatment, only 60.9% (n = 53) received overall treatment meeting our definition of “minimally adequate”.

### Treatment of TRD patients

Four TRD patients dropped out of the study, resulting in 83.3% (n = 20) being evaluated at eight weeks. Most (n = 18; 90%) used ADs, all of whom had a DCG-concordant AD treatment dosage. However, 77.8% (n = 14/18) used AD monotherapy. Consequently, as only four TRD patients used AD combinations, and one received augmentation using quetiapine, this resulted in 25% (n = 5) of all TRD patients receiving DCG-recommended pharmacological treatment for depression. Compared with the non-TRD patients, a significantly smaller proportion received adequate pharmacotherapy for their depression (25% vs. 83.9%, *p* < 0.005).

Altogether 20% (n = 4) of the 20 TRD patients evaluated at eight weeks had received treatment meeting the criteria for “minimally adequate,” i.e., having a DCG-concordant AD dosage at eight weeks, using pharmacological augmentation or an AD combination, and having had two or more follow-up visits at the treating facility. Overall treatment adequacy was therefore significantly lower (p = 0.002) than in non-TRD patients.

### Patient outcomes

**Fig. 1:**
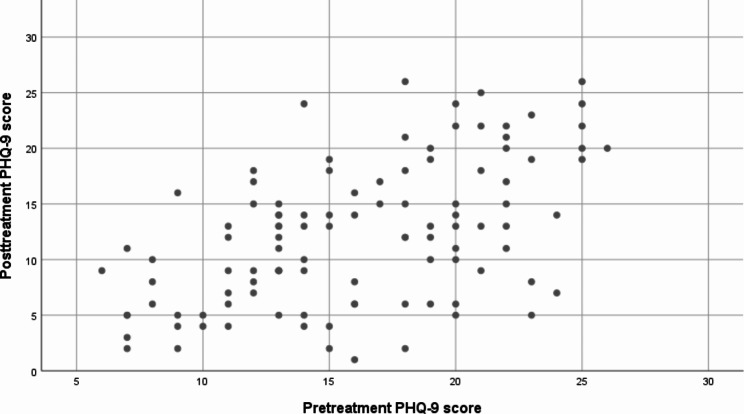
Scatter plot of PHQ-9 scores before and after treatment in psychiatric patients with depressive disorders. PHQ-9, Patient Health Questionnaire

Our primary outcome was the within-individual change in PHQ-9 scores (Fig. 1). A significant difference (*p* < 0.0005) of 3.8 points emerged between PHQ-9 scores at baseline and eight weeks. At eight weeks, 10.3% (n = 11) of patients remaining in the study had a PHQ-9 score of ≤ 4 points, considered full remission, and 39.3% (n = 42) had a score of < 10 points, indicating subthreshold symptoms or remission.

**Fig. 2:**
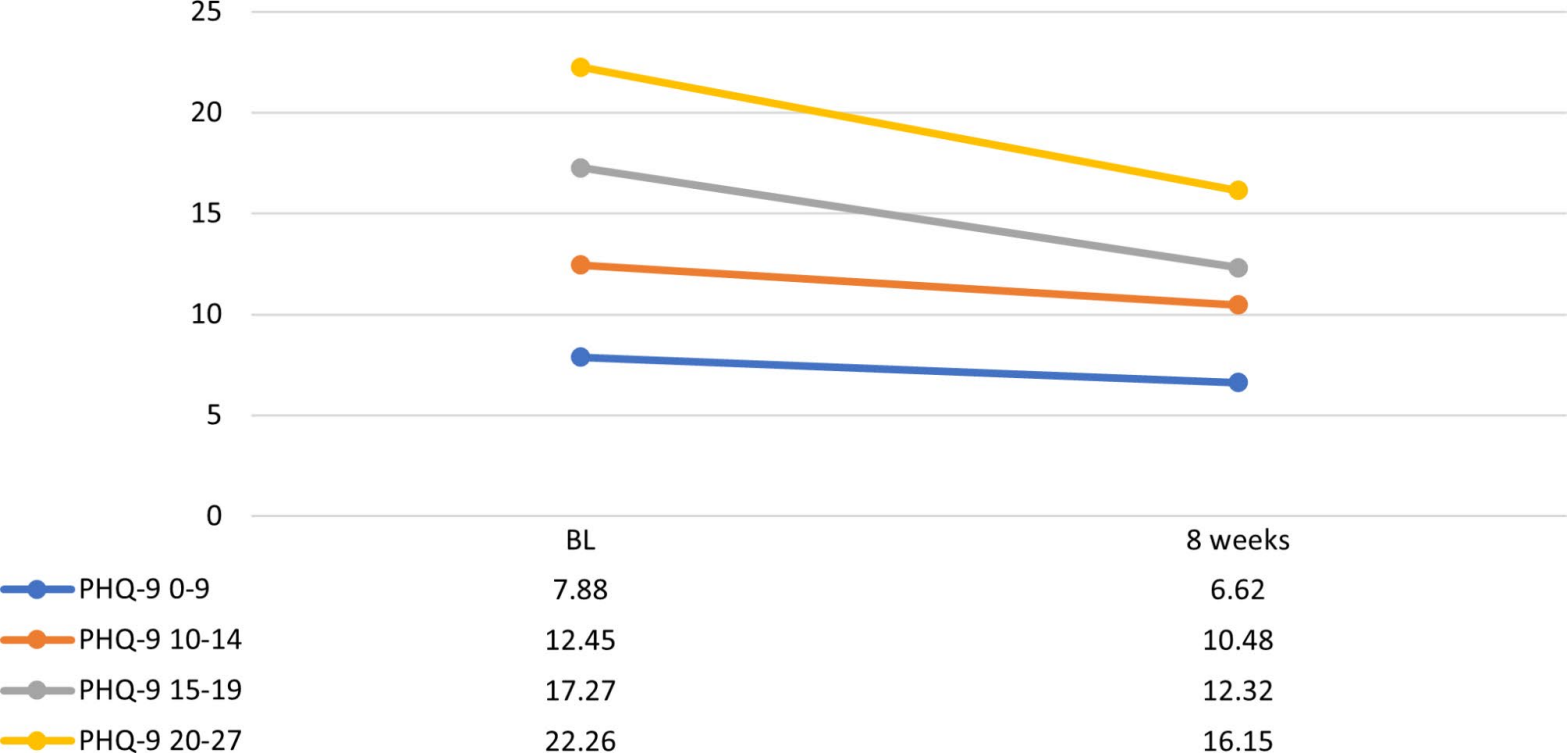
Change in PHQ-9 scores in psychiatric patients with depressive disorders. Group division based on baseline depression severity. Y-axis: PHQ-9 scores, X-axis: time points at baseline (BL) and 8 weeks. PHQ-9, Patient Health Questionnaire; PHQ-9 0–9, mild depression; PHQ-9 10–14, moderate depression; PHQ-9 15–19, moderately severe depression; PHQ-9 ≥ 20, severe depression

Self-report scale results are presented in Table 4. Mean PHQ-9 scores at baseline indicated moderately severe depressive symptoms (16 ± 5.1), decreasing to a mild level (12.2 ± 6.3) over the eight-week follow-up (*p* = 0.005). However, when patients were divided into groups according to baseline depression severity, wide variations emerged. At baseline, 12.5% (n = 16) reported subthreshold depressive symptoms (PHQ-9 < 10), 31.3% (n = 40) reported mild symptoms (PHQ-9 10–14), 28.9% (n = 37) reported moderate symptoms (PHQ 15–19), and 27.3% (n = 35) reported severe symptoms (PHQ-9 **≥** 20). The PHQ-9 score change at eight weeks reached statistical significance for all baseline depression severity groups with a baseline PHQ-9 score ≥ 10 (Fig. 2).

**Table 4:** Self-report scale results of psychiatric patients (n = 107) with depressive disorders at baseline and eight weeks

	Baseline, n = 128 Mean (SD)	Eight weeks, n = 107 Mean (SD)	Change (8 − 0 weeks) Mean (SD)	*p*-value*
**PHQ-9**	16.0 (5.1)	12.2 (6.3)	-3.8 (5.7)	*p* < 0.0005
**Oasis**	12.0 (3.7)	9.9 (4.4)	-2.0 (3.8)	*p* < 0.0005
**SDS**	21.1 (6)	17.9 (7)	-3.4 (7.1)	*p* < 0.0005

PHQ-9, Patient Health Questionnaire; Oasis, Overall Anxiety and Impairment Scale; SDS, Sheehan Disability Scale; SD, Standard deviation

*Baseline vs. eight-week time point, Paired samples T-test

We ran a multivariate logistic regression model to ascertain the effects of eight independent variables (age, sex, baseline values of PHQ-9, OASIS, AUDIT, and MSI, the variables indicating either first episode or recurrent depression, and AD dosage as either adequate or not) on the likelihood of reaching a PHQ-9 value < 10. The model was statistically significant, X^2(8) = 31.080, *p *< 0.005, explaining 39.2% of the variance and correctly classifying 79.6% of the cases (sensitivity 62.5%, specificity 88.5%, PPV 74.1%, and NPV 81.8%). Among the independent variables, only three were statistically significant: baseline PHQ-9 (B=-0.251, *p* = 0.001), the variable marking first episode vs. recurrent depression (F32 or F33) (B = 2.904, *p* = 0.009), and sex (B=-1.436, *p* = 0.032). Better odds of reaching a subthreshold level of depression symptoms (PHQ-9 < 10) were associated with a lower PHQ-9 value at baseline (OR 0.778, 95% CI: 0.669–0.906), having a diagnosis of recurrent depression (OR 18.246, 95% CI: 2.038- 163.345), and being female (OR 0.238, 95% CI: 0.064–0.885).

Finally, we examined the effect of treatment adequacy on PHQ-9 score change for TRD and non-TRD patients separately. Despite some numerical differences, no significant PHQ-9 score reduction was seen in favour of adequate treatment in either patient group (*p* = 0.12 for TRD patients and *p* = 0.09 for non-TRD patients).

## Discussion

This study examined current treatment practice of depression, focusing on AD use in adult psychiatry units of Helsinki University Hospital, Finland. At baseline, most study patients had recurrent, moderately severe depression, and 19% had TRD. Roughly one-third had not received any AD treatment for their current MDE before referral to psychiatric care. During the eight-week follow-up, half of all patients received treatment meeting our minimum requirements for medication and follow-up. However, only few TRD patients received DCG-recommended AD combinations or pharmacological augmentation. Despite reported symptom relief and a significant mean decline in PHQ-9 scores, individual score reductions indicated modest treatment outcomes.

### Patient characteristics and treatment history

Our typical patient was an educated female in her late thirties, referred to psychiatric care for her third, moderately severe depressive episode. Most of our study patients were diagnosed with recurrent depression, 19% with TRD, and 20%, predominately first-episode patients, had a chronic index episode with depression lasting two years or longer.

The mean baseline level of depression was less severe than expected. Initially, all patients had a PHQ-9 score ≥ 10. However, the study screening and the baseline evaluation did not always coincide, resulting in treatment initiation before evaluation in some patients. Baseline symptom ratings may also have been affected by pharmacological treatment initiated shortly before referral, causing symptom reductions while waiting for access to specialized psychiatric care.

Comparing patient characteristics with previously reported findings of patients in Finnish psychiatric care settings [40, 54], we find broadly similar sex and age distributions and mean severity of depression. We also see noteworthy differences compared with these previous studies. First, our patients seemed to suffer from a more persistent course of depression. Our study patients had their first depressive episode at a younger age, suffered more likely from recurrent depression, and had a longer symptomatic period of their current MDE before the baseline interview. Second, prior AD use was rarer than in Vuorilehto et al. [40], as nearly one-third of patients had not used any ADs for their current MDE before referral to psychiatric care.

Our finding of prolonged delay in referral to psychiatric care could reflect Finnish DCG recommendations of staging between different levels of care settings. However, it does not justify the lack of treatment trials in primary care. We did not find an explanation, such as a more severe mean level of depression or acute suicidality, for why so many patients were referred to psychiatric care without prior AD treatment. We do not know whether the failure to meet DCG recommendations is related to their not being appropriately conveyed, or to a scarcity of health care resources. We found some patient-related characteristics possibly influencing help-seeking behaviour, e.g., housing type (living alone vs. others) and lower employment rate, compared with earlier findings [40, 54].

While expecting a higher proportion of TRD among patients in psychiatric care, 19% is still somewhat higher than the 11% suggested in a Finnish nationwide register cohort [41], and it is within the range of prevalences found in other recent studies (e.g., [55–59]). Also, as some patients failed to provide information on AD dosing or duration, affecting the identification of prior adequate treatment trials, the actual proportion of TRD may have been higher. Hence, our ratio of TRD patients indicates that those deemed challenging to treat in primary care are at least partly correctly referred to psychiatric care.

### Treatment during follow-up

Depression chronicity and recurrence increase the probability of poor outcomes, both acute and long-term [60–64]. Therefore, characteristics associated with poor outcomes, namely TRD, should alert clinicians to consider treatment enhancement. However, our findings indicate that this may have been overlooked in our study patients’ treatment planning and follow-up.

The results regarding mere AD use or the bare minimum of follow-up visits appear acceptable. However, due to non-implemented DCG-recommended AD combination and augmentation strategies, the observed pharmacological treatment of TRD patients can be considered substandard. Follow-up visits were unevenly distributed among patients. One-third did not meet their psychiatrist in follow-up, yet a few patients received disproportionally many visits overall, which was not explained by depression severity or acute suicidality. Half of all patients and only 20% of TRD patients received treatment considered “minimally adequate”, meaning adequately combined pharmacological treatment and follow-up.

Our findings of current depression treatment indicate a clear gap between guideline recommendations and clinical practice but resonate with earlier results [27, 36, 41, 65]. Nevertheless, the now-observed quality-of-care problems warrant attention. Concerning treatment augmentation strategies, the DCG presents AD combinations or pharmacological augmentation as plausible options when treating TRD in psychiatric care. However, as patients are referred from primary to psychiatric care in severe situations requiring expertise, we consider the expectation of using AD combinations or pharmacological augmentation in treating TRD justified. Although some TRD patients may benefit from additional AD monotherapy trials [66], medication augmentation strategies are recommended to enhance outcomes [67–71]. In our study, AD combinations were equally likely to be administered to TRD and non-TRD patients, and augmentation pharmacotherapy was strikingly sparse in both patient groups. We do not know whether symptom rating scales were used during each visit to guide clinicians’ treatment decisions, but clearly, TRD patients did not receive DCG-recommended drug enhancement as a rule. Simply put, a similar treatment regimen was used for all patients, regardless of their medical history.

Explanations for DCG-discordant treatment practice might include unawareness of patients’ symptom and treatment history and, therefore, failure to recognize TRD. Staff shortages may affect the quality of care, and the clinical supervision of doctors in training, decreasing the awareness of DCG recommendations in primary and psychiatric care. Also, patients may be wary of using multiple pharmaceuticals or substances not primarily used for MDD for fear of side effects and stigmatization. However, patients and caregivers must be educated to recognize the risks of prolonged ineffective treatment.

Therefore, proper psychoeducation should not be overlooked as a stepping stone in depression treatment. We need to share information with patients on disabilities and poor outcomes connected to depression chronicity and recurrence, justifying enhanced treatment options when considered necessary.

### Patient outcomes

Overall, symptom rating scores indicated significant improvement in all measured entities. However, measured by PHQ-9 score reductions, only 10% of patients reached depression remission and 30% treatment response. Notably, nearly half of the patients experienced no treatment response. These findings of suboptimal treatment outcomes cannot be overlooked despite the relatively short study duration of eight weeks and the uncertainty of effective dose adjustment for a maximum response during follow-up.

The finding of a substantial proportion of non-responders may relate to clinical characteristics, such as depression chronicity in the form of a longer symptomatic period, evident at baseline. Chronicity was seen notably in the first-episode patients. In contrast, patients with recurrent depression showed faster initiation of treatment and better treatment results. Our result suggests an accentuation of chronicity compared with earlier findings from a Finnish psychiatric care cohort [54]. In this earlier study, treatment response was mainly seen within six months of treatment initiation in psychiatric care, after which recovery was sparse. Other studies also stress the effectiveness of treatment early in illness [63, 72], that a greater illness burden reflects on the treatment required, and that results diminish for each treatment step needed [60, 66].

We observed PHQ-9 scores decreasing more between baseline and the two-week time point than in the last six weeks of follow-up. The reason was not clarified but may relate to an unspecified reaction to treatment initiation. However, as early treatment response has been associated with better outcomes [16, 73], the possible predictive nature of this clinical factor should be noted in follow-up. Nevertheless, this finding highlights the importance of repeated symptom and response tracking throughout the first weeks and months of acute depression treatment to ensure treatment intensification if needed.

### Study strengths and limitations

This study aimed at a representative sample of patients with depressive disorders referred to psychiatric care with a PHQ-9 score ≥ 10 and a new AD initiated. Detailed data were collected during an eight-week prospective study using in-person interviews and standardized evaluation scales. This study highlights the flow of patients from primary to specialized psychiatric care and follows patients in usual treatment. Therefore, we describe current, real-life patient characteristics and depression treatment strategies used in psychiatric care.

There are some limitations to consider. First, the number of patients recruited was moderate (n = 131). Second, we did not collect data on the number of patients declining participation or the reason for this, both limitations affecting the generalizability of the results. However, given the similarity of characteristics between our cohort and other comparable psychiatric cohorts within the Helsinki University Hospital area [40, 54], we find it unlikely that this would have resulted in a marked selection bias concerning the AD treatment provided to our study patients. In our view, possible selection bias may have enriched adherent doctors and patients, and patients without characteristics necessitating urgent clinical measures such as imminent suicide risk. Also, similar patient characteristics and suboptimal treatment practices have been reported in a recent nationwide register-based cohort study [41], further supporting our findings. Third, data collection on the prior course of illness and treatment received relied on patients’ recollections, a source for possible data inaccuracy. For example, missing data on AD dosing and duration of use affected the recognition of adequate treatment trials, likely resulting in a slight underestimation of TRD prevalence. Fourth, despite longitudinal follow-up, data collection focused on the current cross-sectional state of two and eight weeks, limiting the information available on daily medication adherence, planned and realized dose changes between research time points. Therefore, even if meeting the therapeutic dose criteria of the DCG, the AD dosage could be suboptimal at an individual level. Finally, we also compared outcomes between patients with adequate vs. inadequate treatment and found no significant difference. However, this was a non-randomized, observational study from which causal inferences from the role of treatment on the observed outcome are not justified.

## Conclusion

Our results suggest that inadequate treatment of depression continues to occur in psychiatric care settings. Observed treatment outcomes were modest, and only 10% of patients reached remission. AD treatment was lacking particularly for TRD patients, as only 25% received DCG-recommended AD combinations or pharmacological augmentation. The intensity of treatment monitoring was inadequate for one-third of all patients.

Considering the escalating health burden caused by depressive disorders impacting the economy and personal loss, these findings of suboptimal treatment practices warrant attention. Based on our findings, we stress the importance of structured data collection and use of MBC to ensure quality of all treatment for depression.

## Data Availability

The datasets generated and analysed during the current study are not publicly available due to restrictions imposed by the Finnish data protection legislation and research permits but are available from the corresponding author on reasonable request.

## Abbreviations

AD: Antidepressant
AUDIT: The Alcohol Use Disorders Identification Test
C-SSRS: The Columbia–Suicide Severity Rating Scale
DCG: Depression care guideline
FTND: The Fagerström Test for Nicotine Dependence
ICD-10: International Statistical Classification of Diseases and Related Health Problems 10th Revision
MBC: Measurement-based care
MDD: Major depressive disorder
MDE: Major depressive episode
MDQ: The Mood Disorder Scale
MSI-BPD: The McLean Screening Instrument for Borderline Personality Disorder
OASIS: The Overall Anxiety and Impairment Scale
PHQ-9: The Patient Health Questionnaire-9
SDS: The Sheehan Disability Scale
SHAPS: The Snaith-Hamilton Pleasure scale
TRD: Treatment resistant depression
